# Ethanolic *Gracilaria fisheri* Extract and Purified N-Benzylcinnamamide Inhibit *Staphylococcus epidermidis* Adhesion and Biofilm Formation on Device-Relevant Surfaces

**DOI:** 10.3390/microorganisms14030700

**Published:** 2026-03-20

**Authors:** Kulwadee Karnjana, Sakun Thala, Kanokpan Wongprasert

**Affiliations:** 1 School of Medicine, Walailak University, 222 Thaiburi, Thasala District, Nakhon Si Thammarat 80160, Thailand; kulwadee.kr@wu.ac.th; 2 Department of Anatomy, University of Medicine 2, Khaymar Thi Road, North Okkalapa Township, Yangon 11031, Myanmar; sakunjp@gmail.com; 3 Department of Anatomy, Faculty of Science, Mahidol University, Rama VI Road, Bangkok 10400, Thailand

**Keywords:** *Staphylococcus epidermidis*, antibiofilm, adhesion, medical device, N-benzylcinnamamide, *Gracilaria fisheri*

## Abstract

*Staphylococcus epidermidis* is a leading opportunistic pathogen in medical device-associated infections due to its ability to adhere to abiotic materials and develop biofilms that are difficult to eradicate. This study investigated the antibiofilm potential of an ethanolic extract of the red seaweed *Gracilaria fisheri* and its purified constituent, N-benzylcinnamamide, against *S. epidermidis*. Antibacterial activity was determined, and antibiofilm effects were assessed using the crystal violet assay and confocal laser scanning microscopy (CLSM). Early bacterial adhesion on glass and polyurethane (PU) surfaces was measured. The effect on catheter-associated biofilms was evaluated by scanning electron microscopy (SEM). Transcripts of biofilm- and quorum-sensing-associated genes (*icaA* and *luxS*) were assessed by semi-quantitative RT-PCR. Cytotoxicity was evaluated by MTT assay. At 200 µg/mL, biofilm biomass decreased to 48.21 ± 5.52% with the extract and to 36.65 ± 6.82% with N-benzylcinnamamide. CLSM time-course imaging showed delayed biofilm maturation and less consolidated, discontinuous structures. Surface exposure to the extract markedly reduced early attachment on both materials. On PU catheter segments, SEM demonstrated that N-benzylcinnamamide markedly reduced surface coverage and disrupted three-dimensional biofilm architecture. At the molecular level, transcription of *icaA* and *luxS* was reduced. Both the extract and N-benzylcinnamamide showed minimal cytotoxicity in HeLa cells. These findings support further evaluation of these marine-derived agents as candidates for antibiofilm surface treatments to reduce early medical device colonization.

## 1. Introduction

The increasing use of implanted biomedical devices for diagnostic and therapeutic purposes has been accompanied by a rising incidence of device-associated infections [1]. Among the causative organisms, *Staphylococcus epidermidis* is the predominant coagulase-negative staphylococcus implicated in biomaterial-related infections, including those involving central venous catheters, prosthetic joints, intracardiac devices, cerebrospinal fluid shunts, vascular grafts, and artificial heart valves [2,3,4]. In addition to device-related infections, *S. epidermidis* contributes to bloodstream and skin/soft-tissue infections, particularly in healthcare settings [5,6,7].

A key determinant of *S. epidermidis* persistence on device surfaces is biofilm formation, which enables long-term colonization and reduced susceptibility to antimicrobial therapy [8]. Biofilm-embedded cells are protected by an extracellular polymeric matrix that limits antimicrobial penetration and supports community resilience [9]. This matrix is mainly composed of polysaccharide intercellular adhesin (PIA), which is synthesized by enzymes encoded in the icaADBC locus; particularly *icaA* and *icaD* are strongly correlated with PIA production and enhanced biofilm-forming capacity in Staphylococcus species [10,11]. Moreover, biofilm development is also regulated by quorum-sensing systems, such as the *luxS*-dependent pathway, which has been associated with modulation of biofilm-associated gene expression and virulence-related traits in *S. epidermidis* [12,13]. Research into new antimicrobial agents and biofilm-disrupting approaches is essential to address the challenges posed by *S. epidermidis*, as conventional antibiotics frequently fail to clear these resilient infections [14,15]. Alternative approaches, including plant-derived compounds, are gaining attention as promising options for targeting biofilm-forming bacteria [16]. These innovative strategies aim not only to improve treatment outcomes but also to limit the emergence of antibiotic resistance, which remains a major concern in clinical practice [17].

Natural compounds derived from red algae have attracted growing interest because of their antimicrobial and antibiofilm properties [18,19]. *Gracilaria fisheri* is a red alga rich in bioactive constituents, including polysaccharides, phenolic compounds, and other secondary metabolites [20,21]. Extracts from *G. fisheri* have shown both antimicrobial and antibiofilm activities, notably by reducing biofilm formation of *Vibrio harveyi* [22]. Its purified compound, N-benzylcinnamamide, has been reported to reduce biofilm formation, in part, by interfering with quorum-sensing-associated phenotypes in Vibrio spp. [23].

Antimicrobial device strategies, including silver- or antibiotic-impregnated catheters, may reduce early microbial burden but often provide incomplete and short-lived protection and are less effective once mature biofilms are established [24]. Breakthrough infections and resistance selection remain important concerns [24,25]. In parallel, natural products and algal extracts have been reported to attenuate *S. epidermidis* biofilm formation at sub-MIC levels, frequently through anti-adhesion or quorum-sensing-linked effects rather than strong bactericidal activity [26]. However, relatively few studies evaluate such agents on device-relevant substrates while combining structural biofilm analyses with assessment of biofilm-associated gene expression.

The present study focused on the opportunistic pathogen *S. epidermidis* and its biofilm formation on device-relevant substrates (glass, polyurethane, and catheter segments), and further examined early adhesion, biofilm architecture, and transcription of *icaA* and *luxS* at sub-MIC levels of the *G. fisheri* ethanolic extract and purified N-benzylcinnamamide.

## 2. Materials and Methods

### 2.1. Bacterial Strains and Culture Conditions

*S. epidermidis* ATCC 12228 (BIOTEC, Pathum Thani, Thailand) and *S. epidermidis* BCC 19563 (TBRC 2051; BIOTEC, Thailand) were used in this study. ATCC 12228 is a well-characterized reference strain, whereas BCC 19563 is recorded in the BIOTEC/TBRC collection as an environmental isolate obtained from fruit (*Eugenia malaccensis*). Both strains were grown in tryptic soy broth (TSB; Difco, Becton Dickinson, Sparks, MD, USA) at 37 °C under aerobic conditions with shaking at 250 rpm. Bacterial growth was monitored by measuring the optical density at 600 nm (OD_600_). *S. epidermidis* ATCC 12228 was used for biofilm, adhesion, CLSM, SEM, and gene-expression experiments. *S. epidermidis* BCC 19563 was included for MIC/MBC testing to broaden antibacterial profiling across strains. For all experiments, overnight cultures were adjusted by serial dilution in fresh TSB to a standardized inoculum of OD_600_ = 0.6 (approximately 9 × 10^8^ CFU/mL).

### 2.2. Preparation of Seaweed Extract and Reference Compounds

The red seaweed *G. fisheri* was obtained from the Surat Thani Coastal Fisheries Research and Development Center (Surat Thani, Thailand), washed with distilled water to remove debris and salts, air-dried, and ground to a fine powder. Dried powder (30 g) was extracted with absolute ethanol (500 mL) using a Soxhlet apparatus for 24 h. The solvent was removed under reduced pressure by rotary evaporation at 60 °C to yield the crude ethanolic extract.

**Figure 1:**
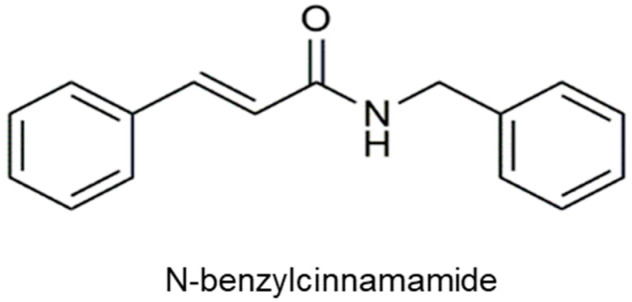
Chemical structure of N-benzylcinnamamide.

N-benzylcinnamamide was purified from the ethanolic extract of *G. fisheri* using a previously described chromatographic procedure [23]. Identity and high purity were confirmed by IR, 1H and 13C NMR, EIMS and HR-TOF-MS, with a purity > 95% by HPLC. The same purified batch was used for all biological assays. The chemical structure was confirmed (Figure 1). The crude extract and purified N-benzylcinnamamide were dissolved in dimethyl sulfoxide (DMSO) to prepare 100 mg/mL stock solutions (5% *v*/*v*) and stored at −20 °C until use. Working solutions were freshly prepared by dilution in the corresponding assay medium.

### 2.3. Determination of Minimum Inhibitory Concentration (MIC) and Minimum Bactericidal Concentration (MBC)

MICs of the *G. fisheri* ethanolic extract and N-benzylcinnamamide against *S. epidermidis* were determined by a broth microdilution assay in flat-bottom 96-well microtiter plates. Briefly, bacterial suspensions were prepared in TSB to a final inoculum of approximately 5 × 10^5^ CFU/mL. Serial dilutions of the extract or N-benzylcinnamamide were prepared in TSB and dispensed into the wells, followed by the addition of the bacterial inoculum. Plates were incubated at 37 °C for 24 h under aerobic conditions. Growth was quantified by measuring absorbance at 595 nm using a microplate reader. The MIC was defined as the lowest concentration that showed no detectable growth compared with the inoculated, untreated control, with uninoculated medium serving as the blank. Rifampicin (5 µg per well) was included as an antibiotic control on each MIC/MBC plate to verify assay performance.

For MBC determination, 100 µL aliquots from wells showing no visible growth were spread on tryptic soy agar and incubated at 37 °C for 24 h. The MBC was defined as the lowest concentration yielding no colony growth or fewer than three colonies on the agar plates.

### 2.4. Bacterial Metabolic Activity (AlamarBlue) Assay

Bacterial metabolic activity was evaluated using the AlamarBlue^®^ assay (Thermo Fisher Scientific, Waltham, MA, USA) according to the manufacturer’s protocol. Briefly, *S. epidermidis* suspensions (9 × 10^8^ CFU/mL) were added to flat-bottom 96-well plates and treated with the *G. fisheri* ethanolic extract or N-benzylcinnamamide at 5–200 µg/mL. Plates were incubated at 37 °C under aerobic conditions. AlamarBlue reagent was then added to each well to a final concentration of 10 µL mL^−1^, followed by incubation for 3 h. Absorbance was measured at 570 nm with 600 nm as the reference wavelength using a microplate reader. Metabolic activity was expressed as the percentage of AlamarBlue reduction relative to untreated controls. The medium containing the corresponding concentrations of extract or compound (without bacteria) was included as a blank control. Sub-MICs (100–200 µg/mL) were then selected for preliminary screening of antibiofilm activity without measurable inhibition of planktonic growth.

### 2.5. Planktonic Growth Kinetics and Viable Counts

To confirm that the concentrations used for biofilm assays did not inhibit planktonic growth, *S. epidermidis* was cultured in TSB containing sub-MICs of the *G. fisheri* ethanolic extract or N-benzylcinnamamide, or in TSB alone (untreated control). Vehicle controls contained TSB with the same final DMSO concentration as treatment conditions. Cultures were incubated at 37 °C under aerobic conditions with shaking at 250 rpm. Growth kinetics were monitored by measuring optical density at 600 nm at 2 h intervals for 24 h.

For viable counts, aliquots were collected at 24 h, serially diluted in sterile saline, and plated (100 µL) onto TSA. Plates were incubated at 37 °C for 24 h, and colonies were quantified. Viable bacteria were expressed as CFU/mL.

### 2.6. Evaluation of Anti-Biofilm Activity

The effect of the *G. fisheri* ethanolic extract and N-benzylcinnamamide on *S. epidermidis* biofilm formation was evaluated under static conditions in TSB. Overnight cultures were adjusted to OD_600_ = 0.6 (approximately 9 × 10^8^ CFU/mL) and diluted 1:1000 in fresh TSB to prepare the working inoculum. Aliquots were added to 96-well plates and co-incubated with sub-MICs of the compounds (100 and 200 µg/mL). Rifampicin (10 µg/mL) and the QS inhibitor furanone C-30 (1.3 µg/mL, ~5 µM) were used as positive controls for biofilm inhibition. Vehicle control wells contained TSB with DMSO at the same final concentration used in the treatment conditions. Plates were incubated at 37 °C without agitation, and biofilms were allowed to develop for 2, 6, 12, 18, or 24 h.

Biofilm biomass was quantified by crystal violet staining. At each time point (2, 6, 12, 18, and 24 h), planktonic cells were removed, and wells were gently washed twice with phosphate-buffered saline (PBS) to eliminate non-adherent bacteria. The remaining biofilms were stained with 0.3% (*w*/*v*) crystal violet for 15 min at room temperature. Excess stain was removed by rinsing with PBS, and plates were air-dried. Bound crystal violet was solubilized with 33% (*v*/*v*) acetic acid (220 µL per well), and absorbance was measured at 570 nm using a microplate reader.

### 2.7. Bacterial Adhesion Assay

Early *S. epidermidis* adhesion was evaluated on glass and polyurethane (PU) materials. Round glass coverslips (10 mm diameter) were used as the inert surface. PU materials were prepared by applying polyurethane (100 µL per coverslip) onto glass coverslips, air-drying for 2 h, and curing overnight under sterile conditions. For surface treatment, glass or PU-coated coverslips were overlaid with *G. fisheri* extract (200 µg/mL; 100 µL per coverslip), air-dried, and cured overnight under the same conditions.

Overnight bacterial cultures were adjusted to OD_600_ = 0.6 (approximately 9 × 10^8^ CFU/mL), diluted 1:1000 in fresh TSB, and 500 µL of the bacterial suspension was added to each well of a 24-well plate containing a coverslip. Plates were incubated statically at 37 °C for 2 h to allow early attachment. Coverslips were then gently washed twice with PBS to remove non-adherent cells and processed either for fluorescence microscopy or for CFU count. All conditions were initiated with the same inoculum density.

For fluorescence imaging, adherent bacteria were stained with DAPI for 30 min in the dark, washed twice with PBS, and mounted using ProLong™ antifade reagent (Thermo Fisher Scientific, Waltham, MA, USA). Images were acquired at 40× magnification using identical exposure settings across all conditions.

To quantify viable adherent bacteria, washed coverslips were transferred to sterile tubes containing 1 mL PBS. Surface-associated cells were detached by sonication for 10 min, followed by vortexing for 2 min. The resulting suspensions were serially diluted in PBS, plated on tryptic soy agar, and incubated at 37 °C for 24 h. Colonies were counted and reported as CFU/mL.

### 2.8. Confocal Laser Scanning Microscopy (CLSM)

To examine the effect of the *G. fisheri* ethanolic extract on *S. epidermidis* biofilm development and maturation, biofilms were grown in 24-well plates under static conditions in the presence of sub-MICs of the extract (200 µg/mL) and N-benzylcinnamamide (200 µg/mL). Rifampicin (10 µg/mL) and furanone C-30 (1.3 µg/mL) were included as reference antibiofilm controls, and untreated cultures served as controls. At 2, 6, 12, 18, and 24 h, planktonic cells were removed, and biofilms were gently washed twice with PBS. Rifampicin was included as an antibiotic comparator to benchmark maximal biofilm reduction in a device-related biofilm model [27], and furanone C-30 was used as a quorum-sensing interference reference to aid interpretation of *luxS*-associated responses [28,29].

Biofilms were stained with wheat germ agglutinin (WGA) and propidium iodide (PI) in PBS (500:1:1, *v*/*v*/*v*) for 15 min at room temperature in the dark. After staining, wells were washed twice with PBS and mounted using ProLong™ antifade reagent. Biofilm architecture was imaged using a confocal laser scanning microscope (FLUOVIEW FV10i, Olympus, Tokyo, Japan). Images were collected to qualitatively compare biofilm architecture over time, including surface coverage, structural organization, and apparent thickness across treatment conditions.

### 2.9. Scanning Electron Microscopy (SEM) Analysis

Based on its defined chemical identity and stronger antibiofilm activity in our screening assays at sub-MICs, N-benzylcinnamamide was selected for established catheter-biofilm SEM and for analysis of biofilm-associated transcriptional responses. SEM was used to examine biofilm surface coverage, architecture, and bacterial morphology on polyurethane catheter segments. Briefly, biofilms were established on sterile catheter segments (~7 mm) by incubating the segments with a standardized *S. epidermidis* suspension (OD_600_ ≈ 0.6; approximately 9 × 10^8^ CFU/mL) at 37 °C for 24 h under static conditions. Segments were then washed three times with PBS to remove non-adherent cells and incubated for an additional 24 h with N-benzylcinnamamide (200 µg/mL), rifampicin (10 µg/mL), or furanone C-30 (1.3 µg/mL). Untreated segments served as biofilm controls.

Following treatment, catheter segments were fixed in 2.5% (*v*/*v*) glutaraldehyde at 4 °C overnight, rinsed with PBS, and post-fixed in 1% (*w*/*v*) osmium tetroxide. Samples were dehydrated through a graded ethanol series, dried by critical point drying, mounted on aluminum stubs, and sputter-coated with gold. Biofilms were imaged using a field-emission scanning electron microscope (Hitachi SU3500, Tokyo, Japan) operated at 5 kV.

### 2.10. Semi-Quantitative RT-PCR Analysis of Biofilm-Associated Gene Expression

*S. epidermidis* biofilms were established for gene-expression analysis by diluting overnight cultures (OD_600_ ≈ 0.6; approximately 9 × 10^8^ CFU/mL) 1:100 in fresh TSB to obtain ~9 × 10^6^ CFU/mL. Aliquots were incubated under static conditions at 37 °C for 24 h to allow biofilm formation in the presence of N-benzylcinnamamide (10, 50, or 100 µg/mL) or furanone C-30 (1.3 µg/mL). Untreated biofilms served as controls. After incubation, planktonic cells were removed, and wells were gently washed twice with PBS. Biofilm-associated cells were then collected for RNA extraction.

Total RNA was isolated using TRIzol™ reagent (Thermo Fisher Scientific) according to the manufacturer’s protocol. RNA concentration and purity were assessed using a NanoDrop™ spectrophotometer (Thermo Fisher Scientific, Waltham, MA, USA). Complementary DNA (cDNA) was synthesized from total RNA using a first-strand cDNA synthesis kit. Semi-quantitative RT-PCR was performed to analyze transcripts.

PCR reactions (25 µL) were performed using gene-specific primers (Table 1). PCR products were resolved on 1.5% agarose gels, stained with ethidium bromide, and visualized using a gel documentation system. Band intensities were quantified by densitometry using ImageJ (version 1.53k; National Institutes of Health, Bethesda, MD, USA) and normalized to 16S rRNA; this approach supports relative comparisons rather than absolute fold-changes.

### 2.11. Cytotoxicity Assessment by MTT Assay

HeLa cells were seeded in 96-well plates at 1 × 10^4^ cells per well and incubated overnight to allow attachment. Cells were then treated with the *G. fisheri* ethanolic extract or N-benzylcinnamamide (0.1–1000 µg/mL) for 48 h. Vehicle controls contained DMSO at ≤0.2% (*v*/*v*), maintained at the same final concentration across all wells. A 10% (*v*/*v*) DMSO treatment was included as a positive control for cytotoxicity. Following treatment, MTT reagent (0.5 mg/mL in serum-free medium; 100 µL per well) was added, and plates were incubated for 4 h at 37 °C in the dark. Formazan crystals were solubilized with DMSO, and absorbance was measured at 570 nm using a microplate reader. Cell viability was expressed as a percentage of the vehicle-treated control.

### 2.12. Statistical Analysis

All experiments were performed in three independent biological replicates. Data are presented as mean ± standard deviation (SD). Statistical analyses were conducted using GraphPad Prism (version 8; GraphPad Software, San Diego, CA, USA). Comparisons among multiple groups were performed by one-way analysis of variance (ANOVA) followed by Tukey’s multiple-comparison test. Growth-curve data were analyzed by two-way ANOVA (treatment × time) with Tukey’s multiple comparisons. Differences were considered statistically significant at *p* < 0.05.

## 3. Results

### 3.1. Antibacterial Potency of G. fisheri Ethanolic Extract and Purified N-Benzylcinnamamide

Antibacterial activity of the *G. fisheri* ethanolic extract and its purified constituent N-benzylcinnamamide against *S. epidermidis* BCC 19563 and ATCC 12228 was determined by broth microdilution (Table 2). The extract inhibited growth with MICs of 12.5 mg/mL (BCC 19563) and 25 mg/mL (ATCC 12228), and corresponding MBCs of 25 and 50 mg/mL (MBC/MIC = 2). In contrast, N-benzylcinnamamide showed MICs of 12.5 and 25 mg/mL, with MBCs equal to the MICs for both strains (MBC/MIC = 1), suggesting greater bactericidal activity than the crude extract.

### 3.2. Sub-MICs Used for Antibiofilm Assays Do Not Affect Planktonic Growth or Viability

**Figure 2:**
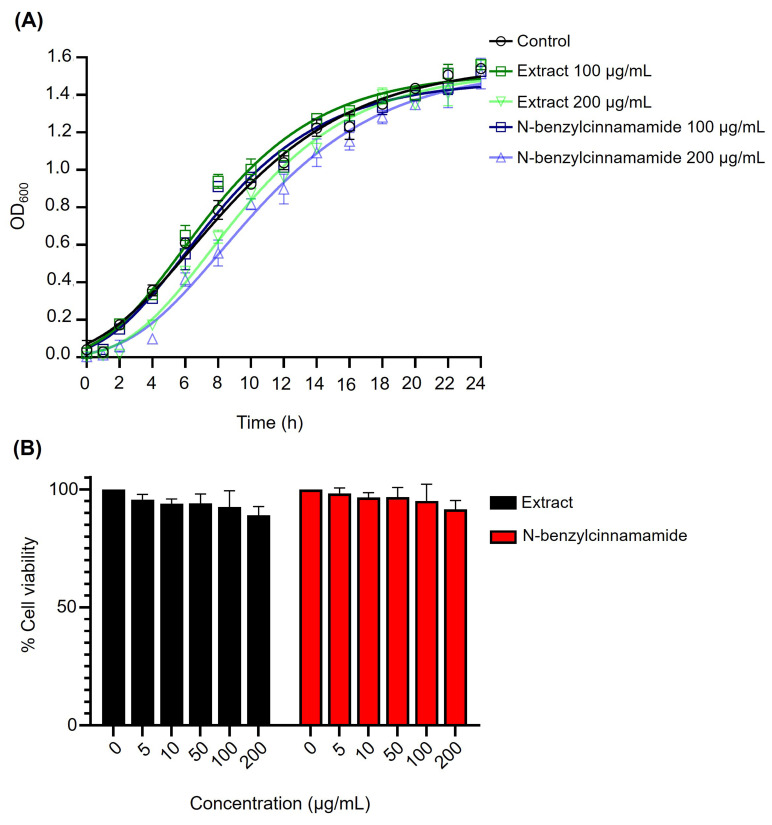
Kinetic growth and viability of *S. epidermidis* after treatment with sub-MICs of ethanolic extract and N-benzylcinnamamide. (**A**) Growth curves (24 h) in the presence of the extract or N-benzylcinnamamide (100 and 200 µg/mL). Statistical analysis was performed using two-way ANOVA; no significant differences were detected (*p* > 0.05). (**B**) Bacterial metabolic activity assessed by AlamarBlue assay following treatments with indicated concentrations (5–200 µg/mL). Data are presented as mean ± SD (*n* = 3).

To verify that the concentrations used in subsequent antibiofilm assays did not inhibit planktonic growth, *S. epidermidis* was exposed to sub-MIC levels (100 and 200 µg/mL) of the *G. fisheri* extract or N-benzylcinnamamide. Growth kinetics monitored over 24 h showed no significant differences in OD_600_ between treated and control cultures (Figure 2A). Consistently, AlamarBlue assays indicated no reduction in bacterial metabolic activity across 5–200 µg/mL for either agent (Figure 2B). Viable counts at 24 h further supported the absence of growth inhibition, with comparable CFU/mL in the control (4.0 × 10^9^), extract-treated cultures (3.6 × 10^9^ at 200 µg/mL), and N-benzylcinnamamide-treated cultures (3.8 × 10^9^ at 200 µg/mL). Two-way ANOVA (treatment × time) with Tukey’s multiple comparisons detected no significant differences at any time point (*p* > 0.05), indicating that these sub-MICs did not measurably affect planktonic growth or viability (Figure 2).

### 3.3. Biofilm Biomass and Time-Course Under Sub-MIC Exposure

**Figure 3:**
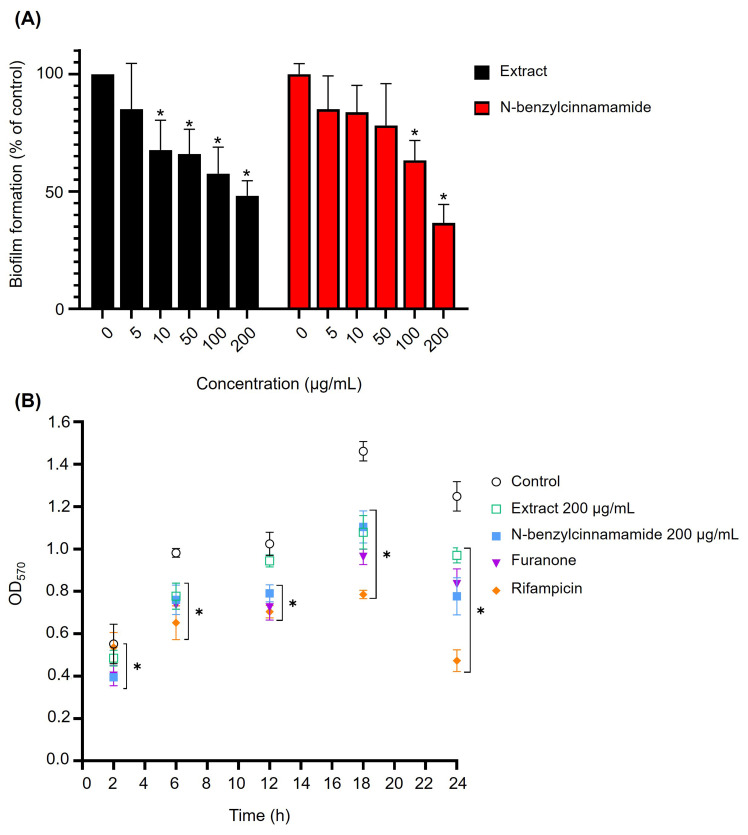
(**A**). Dose-dependent inhibition of biofilm formation by the ethanolic extract and N-benzylcinnamamide in *S. epidermidis*. Biofilm biomass was quantified by crystal violet staining after 24 h and expressed as a percentage relative to untreated control. Data represent mean ± SD (*n* = 3). * *p* < 0.05 versus control. (**B**). Time-course analysis of biofilm biomass formation by *S. epidermidis* in the presence of the ethanolic extract, N-benzylcinnamamide, furanone, and rifampicin. Data represent mean ± SD (*n* = 3). * *p* < 0.05 versus the corresponding control.

Biofilm biomass was quantified by crystal violet staining after exposure to sub-MICs (5–200 µg/mL) of the *G. fisheri* extract or N-benzylcinnamamide. Both agents significantly reduced biofilm biomass in a concentration-dependent manner (Figure 3A). At 200 µg/mL, the extract decreased biomass to 48.21 ± 5.52%, and N-benzylcinnamamide reduced it to 36.65 ± 6.82% of the untreated control.

Time-course analysis further showed sustained suppression of biofilm accumulation over 24 h compared with untreated controls. Notably, N-benzylcinnamamide at 200 µg/mL showed an inhibitory effect comparable to that of the reference quorum-sensing inhibitor, furanone (1.3 µg/mL), at later time points. Rifampicin produced the strongest inhibition at the maturation stage (24 h) (Figure 3B).

### 3.4. Confocal Laser Scanning Microscopy of Biofilm Architecture Under Extract Treatment

**Figure 4:**
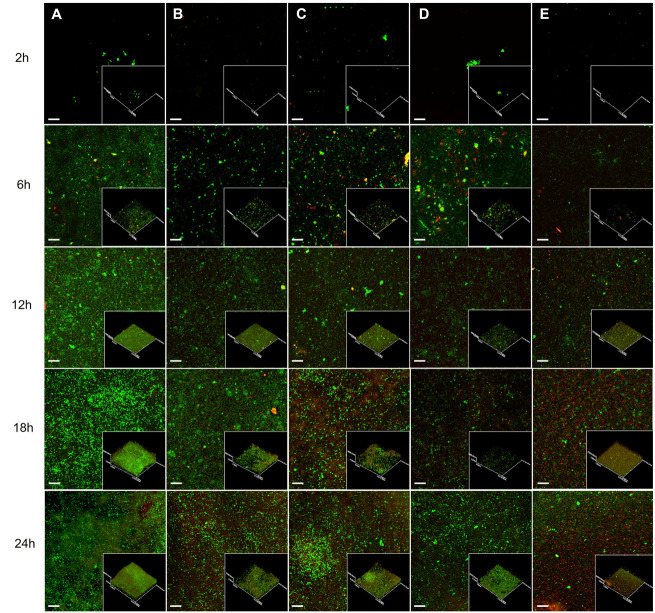
Time-dependent CLSM analysis of biofilm development in *S. epidermidis* treated with the extract. Biofilms were stained with wheat germ agglutinin (WGA, green) and propidium iodide (PI, red) and imaged at 2, 6, 12, 18, and 24 h. (**A**) control, (**B**) 200 µg/mL extract, (**C**) 200 µg/mL N-benzylcinnamamide, (**D**) furanone (1.3 µg/mL), and (**E**) rifampicin (10 µg/mL). Scale bar = 20 µm.

Biofilm development was examined by CLSM over 2–24 h in the presence of *G. fisheri* extract (100 or 200 µg/mL) and reference antibiofilm controls (furanone and rifampicin) (Figure 4). In untreated cultures, *S. epidermidis* progressed from early surface-associated clusters (2 h) to dense microcolonies (6 h) and a multilayered, mature biofilm by 18–24 h (Figure 4A). Both WGA and PI signals increased over time, and PI positivity was detectable by 6 h, consistent with the presence of a subpopulation of membrane-compromised cells within developing microcolonies. Although overall surface coverage at 18 h appeared broadly similar between groups, extract-treated biofilms showed reduced microcolony density and a less compact, more discontinuous architecture, consistent with the corresponding reduction in crystal violet biomass.

Extract exposure delayed biofilm progression in a concentration-dependent manner. At 100 µg/mL, biofilms formed but remained less compact, with reduced microcolony density and a looser architecture at later time points (Figure 4B). At 200 µg/mL, disruption was more pronounced, with more discontinuous coverage and an overall thinner, weakly consolidated structure at 18–24 h (Figure 4C). Furanone produced a similarly fragmented biofilm architecture (Figure 4D), whereas rifampicin resulted in minimal surface-associated structures across all time points (Figure 4E). These imaging data corresponded with the biomass assays and indicated that the extract impairs biofilm maturation and structural consolidation, with greater effects at 200 µg/mL.

### 3.5. Early S. epidermidis Adhesion on Glass and Polyurethane Surfaces

**Figure 5:**
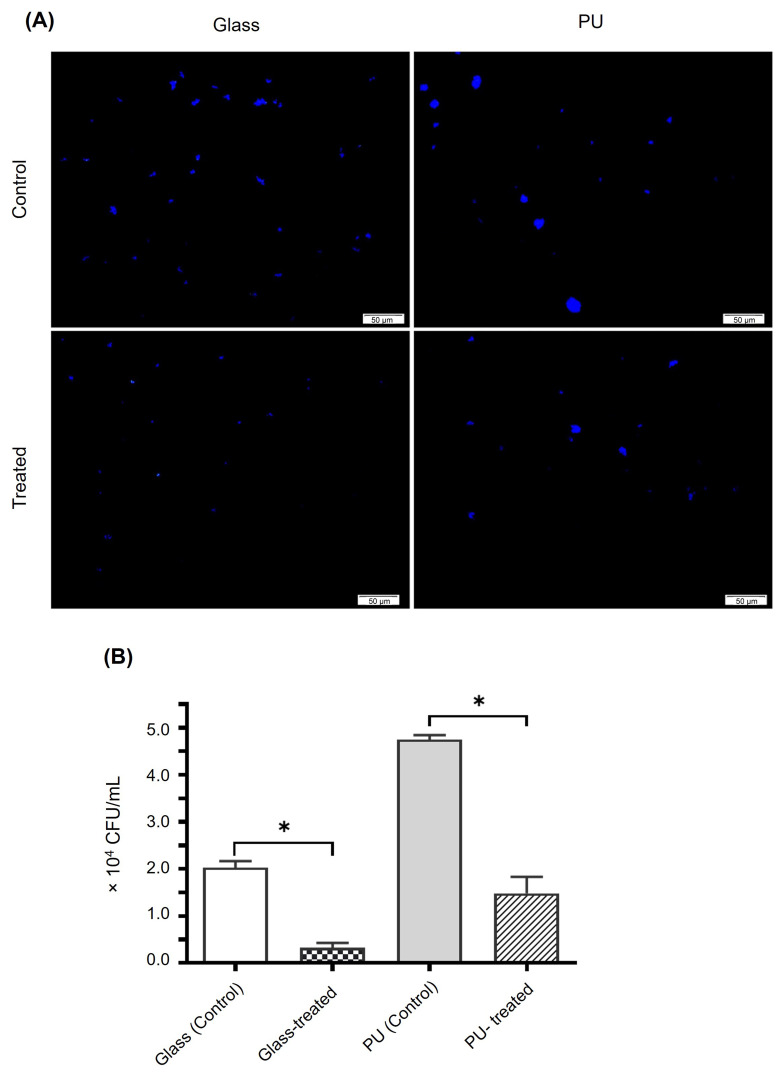
(**A**). Early adhesion of *S. epidermidis* on glass and polyurethane surfaces. Representative DAPI-stained fluorescence images (40×) showing bacterial attachment on the surfaces (glass and PU). Scale bars = 50 µm. (**B**). Quantification of viable adherent *S. epidermidis* cells on glass and PU surfaces. Data are presented as mean ± SD (*n* = 3). Statistical significance was determined by one-way ANOVA (* *p* < 0.0001 versus the corresponding uncoated control).

Early adhesion of *S. epidermidis* to glass and polyurethane (PU) was assessed after 2 h of static incubation. Fluorescence microscopy revealed clear materials-dependent differences in baseline attachment: uncoated PU supported substantially greater adhesion with dense bacterial clusters, whereas attachment on uncoated glass was more dispersed (Figure 5A).

Surface treatment with the crude *G. fisheri* extract (200 µg/mL) significantly reduced the number of DAPI-stained surface-associated bacteria on both materials compared with the corresponding uncoated controls (*p* < 0.0001) (Figure 5A). Quantitative CFU confirmed these observations (Figure 5B). On glass, extract-treated surfaces reduced adherent bacteria from 2.08 × 10^4^ ± 1.38 × 10^3^ CFU/mL (uncoated control) to 0.33 × 10^4^ ± 1.01 × 10^3^ CFU/mL, corresponding to 84.2% reduction. PU surfaces exhibited higher baseline adhesion (4.70 × 10^4^ ± 9.13 × 10^2^ CFU/mL), which decreased to 1.48 × 10^4^ ± 3.36 × 10^3^ CFU/mL after extract treatment, corresponding to 68.5% reduction.

### 3.6. Established Catheter Surface Biofilms Following N-Benzylcinnamamide Treatment

**Figure 6:**
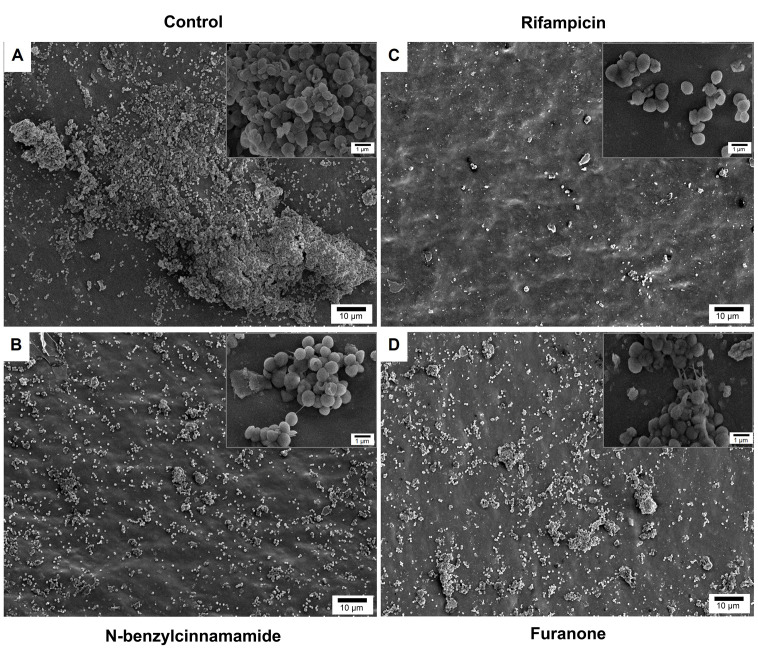
Scanning electron micrographs of established *S. epidermidis* biofilms on polyurethane catheter surfaces after treatment. (**A**) Untreated control. (**B**) N-benzylcinnamamide (200 µg/mL). (**C**) rifampicin (10 µg/mL). (**D**) furanone (1.3 µg/mL). Insets show higher magnification images of bacterial morphology. Scale bars = 50 µm (main images) and 3 µm (insets).

SEM was used to visualize *S. epidermidis* biofilms on polyurethane catheter surfaces following N-benzylcinnamamide treatment (Figure 6). Untreated controls showed extensive surface coverage by dense, three-dimensional biofilm structures with compact bacterial aggregates embedded in extracellular material (Figure 6A). N-benzylcinnamamide (200 µg/mL) markedly reduced biofilm coverage, leaving exposed regions with only scattered microcolonies and small residual clusters (Figure 6B). Rifampicin (10 µg/mL) produced the strongest biofilm inhibition, with predominantly sparse remnants and loss of organized three-dimensional architecture (Figure 6C). Furanone (1.3 µg/mL), similar to that of N-benzylcinnamamide, disrupted biofilm continuity with poorly organized residual structures (Figure 6C). At higher magnification, bacterial cell morphology appeared largely preserved in all treated groups, indicating that these agents primarily disrupted biofilm architecture rather than causing obvious cell deformation.

### 3.7. Transcription of Biofilm and Quorum Sensing Associated Genes (icaA and luxS)

**Figure 7:**
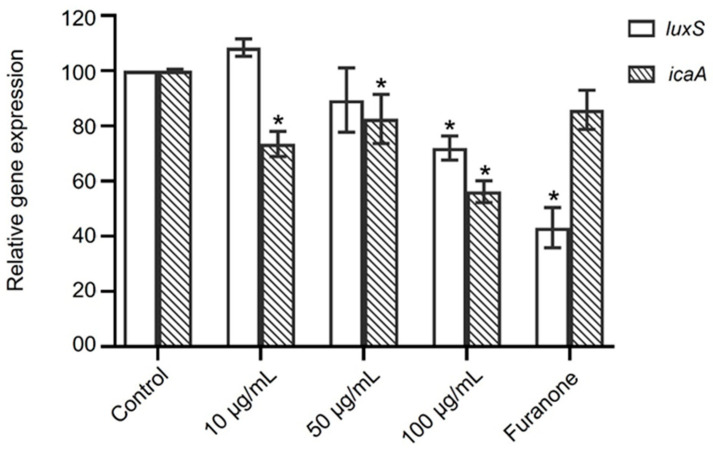
Semi-quantitative RT-PCR analysis of biofilm-associated genes *icaA* and *luxS* transcripts normalized to 16S rRNA. Data represent mean ± SD from three independent experiments. * *p* < 0.05 versus control.

Expression of the biofilm-associated genes *icaA* and *luxS* was assessed in *S. epidermidis* biofilms after exposure to N-benzylcinnamamide (10–100 µg/mL) and normalized to 16S rRNA (Figure 7). N-benzylcinnamamide at 10 and 50 µg/mL produced minor changes in *luxS* transcripts (108.4 ± 3.1% and 89.4 ± 11.7% of control, respectively), whereas *icaA* expression showed a moderate reduction to 73.5 ± 4.6% and 82.6 ± 8.9%, respectively. At 100 µg/mL, N-benzylcinnamamide caused a more pronounced decrease in both genes, lowering *luxS* expression to 72.0 ± 4.3% and *icaA* to 56.2 ± 3.9% of control. In contrast, the antibiofilm reference furanone (1.3 µg/mL) substantially reduced *luxS* expression to 43.2 ± 7.3%, while icaA remained close to control levels (85.8 ± 7.1%).

### 3.8. Cytotoxicity of G. fisheri Extract and N-Benzylcinnamamide in HeLa Cells

**Figure 8:**
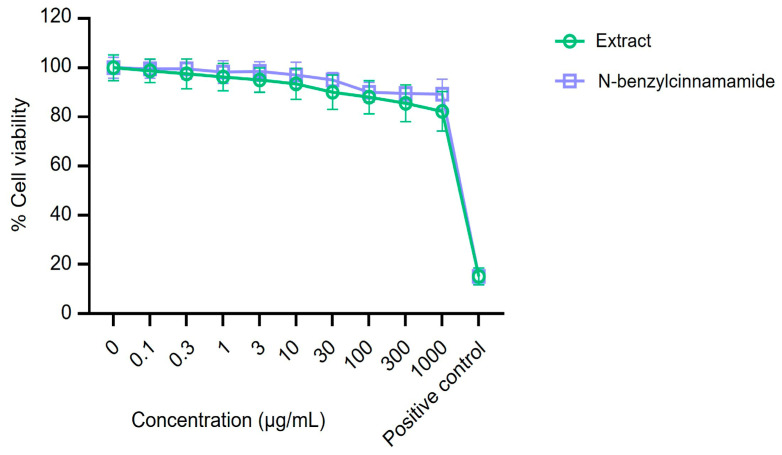
Cytotoxicity of *G. fisheri* ethanolic extract and N-benzylcinnamamide in HeLa cells. Cells were exposed to the extract or N-benzylcinnamamide (0.1–1000 µg/mL) for 48 h, and viability was assessed by MTT assay. Data are presented as mean ± SD (*n* = 8).

Cytotoxicity was assessed by MTT assay in HeLa cells after 48 h exposure to the *G. fisheri* ethanolic extract or N-benzylcinnamamide (0.1–1000 µg/mL). Across the tested concentration range, neither treatment caused a significant reduction in cell viability relative to vehicle controls, with viability remaining >80% (Figure 8). These findings indicate minimal cytotoxicity under the conditions tested and support the use of the extract and N-benzylcinnamamide at concentrations relevant to the antibiofilm and anti-adhesion assays.

## 4. Discussion

Device-associated infections remain a persistent clinical problem, and *Staphylococcus epidermidis* is a major pathogen because it readily adheres to abiotic materials and forms biofilms that are difficult to eradicate [1,30,31]. Here, we demonstrate that an ethanolic extract of *Gracilaria fisheri* and its purified constituent N-benzylcinnamamide attenuate *S. epidermidis* biofilm formation and early adhesion on device-relevant substrates.

MIC/MBC testing showed antibacterial activity of the crude extract, whereas N-benzylcinnamamide was bactericidal (MBC/MIC = 1). The high MIC/MBC values suggest limited potency for systemic antibacterial therapy. This study instead focused on sub-MIC antibiofilm/anti-adhesion effects, as well as localized surface exposure, which are more compatible with surface-directed approaches to limit early device colonization. The antibiofilm and anti-adhesion assays were conducted at sub-MICs (5–200 µg/mL) that did not affect planktonic growth, metabolic activity, or viable counts. Biofilm formation and adhesion were nevertheless reduced, supporting an effect on early attachment and biofilm establishment rather than growth suppression, which is directly relevant to device-associated infections [32,33,34].

Both the extract and N-benzylcinnamamide reduced biofilm biomass in a concentration-dependent manner, and time-course studies indicated sustained suppression of biofilm accumulation during the maturation phase. Under extract exposure, biofilms remained less consolidated, with fragmented microcolonies and visible gaps in coverage at later time points, aligning with the quantitative reduction in biofilm biomass. Across the assays, N-benzylcinnamamide produced the stronger antibiofilm effect. CLSM findings supported a delayed maturation phenotype, although the extract did not completely abolish total surface coverage at 24 h, consistent with impaired progression from initial attachment to a mature biofilm architecture [35,36,37]. Importantly, the PI signal can be observed during normal staphylococcal biofilm development; however, it should not be interpreted as treatment-induced killing in the absence of growth inhibition.

Substrate-based adhesion assays supported device relevance. Polyurethane (PU), a widely used polymer in indwelling devices, showed higher baseline attachment than glass [38,39,40]. Surface treatment with the crude extract substantially reduced adherent CFU on both materials, decreasing attachment to 15.8% of the uncoated control on glass and 31.5% on PU. These reductions suggest the inhibition at the earliest stage of biofilm development. Consistent with these early anti-adhesion effects, SEM analysis of established biofilms on PU catheter segments showed markedly reduced surface coverage and loss of dense three-dimensional structure after N-benzylcinnamamide treatment, leaving only scattered residual microcolonies.

Our data show that sub-MICs of *G. fisheri* ethanolic extract and N-benzylcinnamamide reduce staphylococcal biofilm biomass and disrupt biofilm architecture on device-relevant materials, without measurable inhibition of planktonic growth. These findings are consistent with prior reports that seaweed-derived agents often attenuate biofilms through anti-adhesion and structural modulation rather than direct bactericidal activity. Rima et al. reported that extracts from green (*Ulva lactuca*), brown (*Stypocaulon scoparium*), and red (*Pterocladiella capillacea*) seaweeds (50 µg/mL) reduced *Staphylococcus aureus* biofilm burden by approximately 1–2 log CFU/mL relative to untreated controls, and reduced biofilm density and matrix staining [26]. In that study, the dichloromethane extract of *U. lactuca* primarily impaired surface adhesion and early biofilm development rather than exerting overt bactericidal effects [26]. Shanmuganathan et al. further showed that silver nanoparticles synthesized using an aqueous extract of the red seaweed *Gracilaria verrucosa* were active against multidrug-resistant *S. aureus* (MIC 3.8 µg/mL) and disrupted established biofilms [41]. Taken together, these studies and our results support seaweeds, particularly red seaweed, as a valuable source of antibiofilm agents that weaken staphylococcal biofilms through anti-adhesion activity and disruption of biofilm architecture, with variable effects on planktonic growth.

Biofilm formation requires two sequential steps: cell adhesion to a solid substrate, followed by cell–cell adhesion, resulting in multiple layers of cells [10,42]. These processes are tightly regulated at the genetic level, particularly by the *ica* locus, which controls production of polysaccharide intercellular adhesin (PIA), and by quorum-sensing pathways that modulate biofilm-associated gene expression [11,43]. The *ica* operon encodes enzymes responsible for synthesizing PIA, a cationic polysaccharide that mediates the intercellular aggregation (accumulation phase) of bacteria, which is critical for forming mature, three-dimensional biofilm structures [11,43,44]. The relationship between LuxS/AI-2 and ica/PIA in *S. epidermidis* has been reported to differ by strain and conditions. Xue et al. [45] reported that exogenous AI-2 increased biofilm formation through enhancing transcription of the *ica* operon, suggesting that LuxS/AI-2 can positively couple to *ica* under some conditions.

The gene-expression changes were closely aligned with structural phenotypes. At concentrations that reduced icaA and luxS, biofilm biomass decreased by approximately 40–60%, maturation was delayed, and catheter-surface microcolonies were less organized. Reduced icaA, which supports PIA production and intercellular aggregation, is consistent with diminished matrix accumulation and the thinner, fragmented structures observed. Decreased luxS also suggests attenuation of AI-2-linked regulation, which may contribute to slower biofilm development. Together, these findings are consistent with effects on matrix-associated and quorum-sensing-linked biofilm pathways. In this study, transcription was assessed by semi-quantitative RT-PCR, which indicates relative directional changes; further qRT-PCR validation will provide more precise quantification of expression magnitude.

In our study, furanone C-30, used as a quorum-sensing interference reference, markedly reduced *luxS* with limited effects on *icaA*, supporting its use as a QS-focused comparator. In contrast, the reduction in biofilm biomass by N-benzylcinnamamide was accompanied by decreased *icaA* and *luxS* transcripts, consistent with a multi-pathway antibiofilm effect involving matrix-associated and quorum-sensing-linked regulation. A similar multi-target profile has been reported for cryptotanshinone, which suppresses early biofilm formation and downregulates *icaA*, *luxS,* and additional adhesin-related genes [46].

Cytotoxicity testing further supported feasibility: in HeLa cells, both the extract and N-benzylcinnamamide showed minimal cytotoxicity after 48 h exposure (0.1–1000 µg/mL), with viability remaining above 80% and no clear dose-dependent decline. Although this does not establish clinical safety, it indicates that concentrations relevant to the antibiofilm assays were not overtly cytotoxic under the conditions tested.

Several limitations should be noted. The present work was designed as an initial biological evaluation of whether surface exposure to the extract/compound can reduce early attachment and biofilm development on device-relevant materials. A key limitation of this work is that all experiments were conducted in vitro using static, microtiter-based biofilm models, which do not reflect the dynamic flow, complex fluids, and polymicrobial communities encountered on indwelling catheters and intravascular devices in clinical settings [1,39]. Surface treatment effects were evaluated without physicochemical characterization, and a formal release profile was not investigated. Future studies should therefore incorporate flow-based catheter models and clinically relevant media to confirm activity under conditions that more closely mimic in vivo device environments.

## 5. Conclusions

This study shows that an ethanolic extract of *Gracilaria fisheri* and its purified constituent N-benzylcinnamamide attenuate *Staphylococcus epidermidis* biofilm formation and early adhesion on device-relevant surfaces. Biofilm biomass was reduced at sub-MICs without measurable effects on planktonic growth, and confocal imaging indicated delayed maturation with less consolidated, discontinuous architecture. Surface treatment decreased early attachment to glass and polyurethane, while SEM revealed reduced surface coverage and disrupted architecture of established catheter-surface biofilms following N-benzylcinnamamide treatment. Transcriptional analysis further showed reduced *icaA* expression, with a smaller effect on *luxS*, supporting an effect on biofilm-associated regulation. Both agents showed minimal cytotoxicity in HeLa cells. Collectively, these findings support further evaluation of *G. fisheri*-derived preparations as adjunctive antibiofilm surface treatments to limit early colonization of medical device materials.

## Figures and Tables

**Table 1:** Specific primers used in this study.

Genes	Primer Sequence (Forward 5′-3′) (Reverse 5′-3′)	Annealing Temperature (°C), Time, Cycles	Product Size (bp)
*icaA*	F-ACAGTCGCTACGAAAAGAAA R-GGAAATGCCATAATGACAAC	56 °C, 1 min, 35 cycles	103 bp
*luxS*	F-TTCGTCTAGCCGGGACTATG R-TGTTCCTTATTGGGCTGTTTG	58 °C, 30 s, 40 cycles	84 bp
16sRNA	F-AGAGTTTGATCMTGGCTCAG R-TACGGYTACCTTGTTACGACTT	65.2 °C, 2 min, 35 cycles	1500 bp

**Table 2:** Minimum inhibitory concentration (MIC) and minimum bactericidal concentration (MBC) of ethanolic extract and N-benzylcinnamamide against *S. epidermidis* strains BCC 19563 and ATCC 12228.

Compounds	*S. epidermidis* BCC 19563			*S. epidermidis* ATCC 12228		
	MIC (mg/mL)	MBC (mg/mL)	MBC/MIC	MIC (mg/mL)	MBC (mg/mL)	MBC/MIC
Ethanolic extract	12.5	25	2	25	50	2
N-benzylcinnamamide	12.5	12.5	1	25	25	1

## Data Availability

The original contributions presented in this study are included in the article. Further inquiries can be directed to the corresponding author.

## Abbreviations

The following abbreviations are used in this manuscript:

ATCC	American Type Culture Collection
BCC	BIOTEC Culture Collection
CAUTI	Catheter-associated urinary tract infection
CFU	Colony-forming unit
CLSM	Confocal laser scanning microscopy
DAPI	4′,6-Diamidino-2-phenylindole
DMSO	Dimethyl sulfoxide
EPS	Extracellular polymeric substances
MBC	Minimum bactericidal concentration
MIC	Minimum inhibitory concentration
OD	Optical density
PBS	Phosphate-buffered saline
PI	Propidium iodide
PIA	Polysaccharide intercellular adhesin
PU	Polyurethane
RT-PCR	Reverse transcription polymerase chain reaction
SEM	Scanning electron microscopy/microscope
TSB	Tryptic soy broth
TSA	Tryptic soy agar
WGA	Wheat germ agglutinin
